# The Use of Megamolecular Polysaccharide Sacran in Food and Biomedical Applications

**DOI:** 10.3390/molecules26113362

**Published:** 2021-06-02

**Authors:** Lisa Efriani Puluhulawa, I Made Joni, Ahmed Fouad Abdelwahab Mohammed, Hidetoshi Arima, Nasrul Wathoni

**Affiliations:** 1 Department of Pharmaceutics and Pharmaceutical Technology, Faculty of Pharmacy, Universitas Padjajaran, Sumedang 45363, Indonesia; Lisa20001@mail.unpad.ac.id; 2 Department of Physics, Faculty of Mathematics and Natural Sciences, Universitas Padjajaran, Sumedang 45363, Indonesia; imadejoni@phys.unpad.ac.id; 3 Functional Nano Powder University Center of Excellence (FiNder U CoE) Padjadajaran Universitas Padjajaran, Sumedang 45363, Indonesia; 4 Department of pharmaceutics, Faculty of Pharmacy, Minia University, Minia 61519, Egypt; Ahmed.mohamed1@minia.edu.eg; 5 Daiichi University of Pharmacy, Fukuoka 815-8511, Japan; h-arima@daiichi-cps.ac.jp

**Keywords:** polysaccharide, sacran, *Aphanothece sacrum*

## Abstract

Natural polymer is a frequently used polymer in various food applications and pharmaceutical formulations due to its benefits and its biocompatibility compared to synthetic polymers. One of the natural polymer groups (i.e., polysaccharide) does not only function as an additive in pharmaceutical preparations, but also as an active ingredient with pharmacological effects. In addition, several natural polymers offer potential distinct applications in gene delivery and genetic engineering. However, some of these polymers have drawbacks, such as their lack of water retention and elasticity. Sacran, one of the high-molecular-weight natural polysaccharides (megamolecular polysaccharides) derived from *Aphanothece sacrum* (*A. sacrum*), has good water retention and elasticity. Historically, sacran has been used as a dietary food. Moreover, sacran can be applied in biomedical fields as an active material, excipient, and genetic engineering material. This article discusses the characteristics, extraction, isolation procedures, and the use of sacran in food and biomedical applications.

## 1. Introduction

Polysaccharides are the most used natural polymers in pharmacy, in which they are safe, biocompatible, and biodegradable [1]. Polysaccharides such as chitosan, alginate, carrageenan, and cellulose are the most commonly used in pharmaceutical formulations. However, they suffer from several drawbacks, such as poor elasticity [2] and low water retention [3]. Sacran is a polysaccharide polymer that has a high water retention, high potential to develop, and a better elasticity than other polymers.

Sacran is a polysaccharide derived from cyanobacteria. Cyanobacteria are one of the algae that are also popular with blue-green algae [4] and are diverse in terms of genetics. They are not only spread in freshwater, ocean, and land, but can also be found in extreme ecosystems such as hot springs, hypersaline areas, cold environments, and barren deserts [5].

Cyanobacteria are the only prokaryotes that perform oxygenic photosynthesis as a potential biocatalyst, to directly convert CO_2_ into chemicals and other valuable products [6,7,8]. Cyanobacteria can produce polysaccharides with different functional groups, such as carboxylic acid, sulfate, phosphate, and amino acids that are responsible for adsorption. The texture of the polysaccharide produced by the cyanobacteria looks like a gel. Therefore, it has been a good candidate for the food industry and medicine materials [9]. Reports about the function of polysaccharides produced by freshwater cyanobacteria are still limited. As a result, research about the function of polysaccharides produced by freshwater cyanobacteria has widely been developed [10]. One of the cyanobacteria that live in freshwater is *Aphanothece sacrum* (*A. sacrum*).

*A. sacrum* is a green plant that has long been consumed by the Japanese and grown in Kogane river [9]. *A. sacrum* can produce a polysaccharide called sacran. Sacran has a negative charge in water. Due to its high viscoelasticity and water retention, it has been applied in many cosmetics and medical products [11].

Nowadays, sacran has widely been utilized in biomedical fields due to its high potential compared to other polymers, as well as its expandability, its good water resistance, and its good elasticity. Moreover, sacran can also be used as an active material, additive, and genetic engineering material for wound dressings, an anti-allergy agent, an anti-inflammatory agent, and an agent to deliver drugs to a tumor site. In the biomedical field, sacran has many functions, while, on the other hand, its availability is limited due to the lack of its cultivating method. Therefore, this article explains the extraction method of sacran from *A. sacrum*, as well as its isolation and purification.

## 2. Methodology

**Figure 1:**
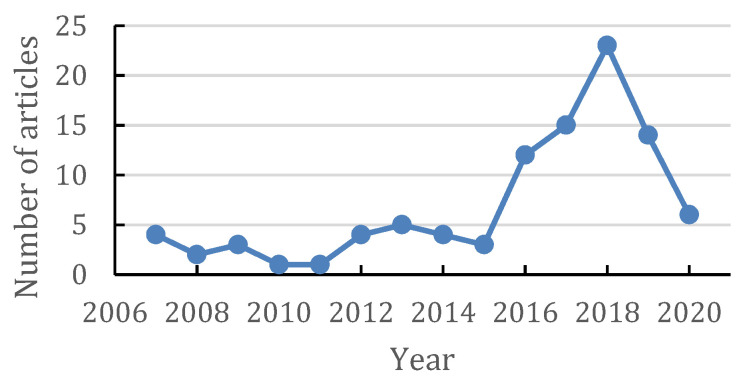
Graphic of total articles used by year.

This article has been written by compiling and reviewing some journals from different websites such as Research Gate, Science Direct, Scopus, and Google Scholar with keywords “Sacran from *Aphanothece sacrum*,” “Sacran polysaccharide,” “*Aphanothece sacrum*,” “Cyanobacteria polysaccharide,” “Sacran activity,” “Anti-allergic of sacran,” “anti-inflammation of sacran,” and “Sacran food,” in the time range from 2006 to 2020, where most of the articles found were published from 2016 to 2019, and the total number of articles per year used in this review is described in Figure 1.

## 3. *Aphanothece sacrum*

### 3.1. Aphanothece sacrum

Cyanobacteria are well known as toxin formers in various water systems all over the world, and they have contributed to the toxicity level of communities as they can release toxic compounds [12,13,14,15,16]. However, much research dealing with the metabolites of cyanobacteria has shown that cyanobacterial elements have biological activities related to human health [16]. It has been identified that Cyanobacteria can produce and secrete polysaccharides with different functional groups, such as carboxylic acid, sulfate, phosphate, and amino acids that are responsible for adsorption [17].

*Aphanothece sacrum* (*A. sacrum*) is a unicellular cyanobacterium found in freshwater. It belongs to seaweed types that can produce a polysaccharide known as sacran [9]. *A. sacrum* is a microalga grown in the Kyushu river, Japan [18], and it has a jelly matrix and a long cluster size [10]. It was classified biologically in the 19th century by Suringar and has been recognized for more than 100 years. However, research dealing with this issue is rarely found as its material availability is low and its cultivating method is less efficient. *A. sacrum* has a higher water content than other popular jellies such as *Nostoc commune*. From this information, it can be inferred that *A. sacrum* has a greater capacity for water retention [10]. It also contains high contents of Ca, Fe, Cu, and Mn that has made it different from other seaweed types [19]. *A. sacrum* is the only microalgae possibly grown as food materials for more than 300 years [9].

### 3.2. Characteristic of Aphanothece sacrum

*A. sacrum* is a prokaryotic alga that does not have chloroplasts to perform photosynthesis, and it has been recognized as a very primitive alga. It is evolutionarily and ecologically close to photosynthetic bacteria. Aphanothece, one of the algae, only has chlorophyll a, not chlorophyll a and b like other higher plants. It is a mediator between photosynthetic bacteria and green plants that have chlorophyll a and ferredoxin chloroplast types, but it resembles photosynthetic bacteria in other particular features [20,21,22].

In the research of Fujishiro et al. [19], the DNA analysis of an isolated *A. sacrum* chain showed that this chain carries two genes of ferredoxin (I and II), an amino acid sequence. In line with that, research undertaken by Wada et al. (1974) has shown that the ferredoxin molecule of *A. sacrum* contains an amino acid that is similar to the ferredoxin of other chloroplast types. The acidic amino acid is more prominent than the basic one. This ferredoxin lacks methionine and tryptophan. It retains one residue of histidine, arginine, and phenylalanine. There are only four cysteine residues found in this ferredoxin, which are also possessed by other plants such as Bumilleriopsis, Equisetum, Zea mays, Cypenis, and Gossypium. Moreover, the results of this research also explained that one molecule of ferredoxin of *A. sacrum* contains two atoms of iron and common volatile sulfur. It also provided the composition of the ferredoxin amino acid of *A. sacrum*.

### 3.3. Sacran

Nowadays, natural polymers are frequently used in several pharmaceutical preparations. One of the natural polymers is sacran, which is derived from *Aphanothece sacrum* [23]. This polysaccharide contains anionic groups such as carboxylate and sulfonate in high concentrations (32% mol of the total sugar) [9,24,25]. Therefore, sacran has a negative charge in water [11]. Sacran is a sulfated natural polymer extracted from the extracellular jelly matrix of *A. sacrum*, a cyanobacterium in freshwater [26]. Sacran contains 11 types of monosaccharides (Glc, Gal, Pria, Xyl, Rha, Fuc, GalA, GlcA, and traces of Ara, GalN, and Mur), with a molecular weight of 1–2.2 × 10^7^ g/mol and a chain length of more than 30 μm [27,28].

Previous research has reported that the physical characters of sacran are unique. It builds gel-like layers that contain water with polyol; 1.3-butanedyol and 1.2-pentanedyol. Its film prevents the penetration by water and chemicals [27]. In solution, sacran is a polyelectrolyte, and because of its electric charge, the conformation of the sacran chain changes depending on its concentration. For instance, a helix transition concentration occurs when a chain of sacran changes from a random coil into a double helix at 0.09%, and gelation concentration occurs when sacran transitions from liquid to gel at 0.25% [28,29]. With increasing concentration, the sacran chain changes into a rigid rod form, showing liquid crystalline (LC) properties [30,31]. Sacran can form a gel-like film that is difficult to re-dissolve in water. It is formed due to polyol 1,3-butilen glycol, which is more superior to skin protection without polyol [32].

Sacran is a heteropolysaccharide composed of various sugar residues. From 11% of the monosaccharides, it contains a sulfate and carboxyl group [28,33]. Sacran is considered a safe biomaterial as *A. sacrum* is a cyanobacterium that has long been used by the people of Kyushu, Japan as a food to treat allergies and gastroenteritis. Sacran can also retain more water than hyaluronic acid or xanthan gum, and it can form hydrogel through the interaction between electrostatic and heavy metal cations [34]. Sacran can orientate itself depending on its concentration. Its structure looks like jelly that can adsorb cationic metals. Moreover, it generates an extracellular jelly matrix that can protect cells [25,35]. Macroscopically, the geometric structure of sacran consists of membrane partitions of three-dimensional cuboid cells to evaporate a uniaxially oriented water solution; thus, it can be developed for medical and pharmaceutical industries [36,37].

### 3.4. Extraction and Isolation of Sacran

*A. sacrum* is a plant that has been cultivated massively in the freshwater of Japan. With metal ions at its extracellular matrix, it forms a jelly-like material, which provides protection to the cellular structure of the algae. The composition of sacran within *A. sacrum* is very high, with 70% of dry *A. sacrum* [38]. Sacran is extracted from *A. sacrum* by evaporating the solvent [39], and this polysaccharide is extracted using a basic solvent generated from the biomaterial that has been washed with acid so that the minerals can be removed [38] 

**Figure 2:**
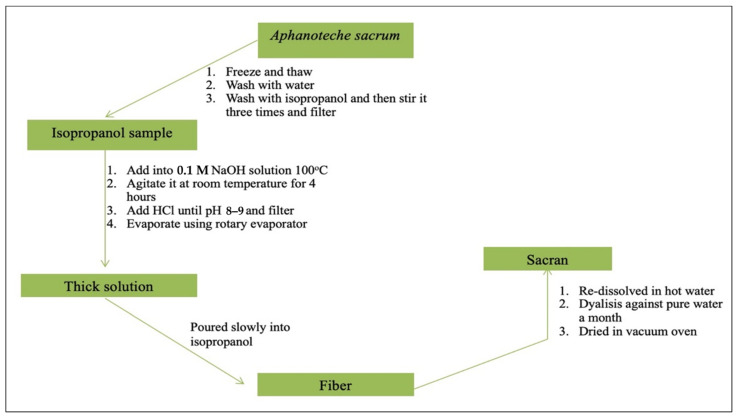
Scheme of sacran extraction process.

A sample of *A. sacrum* is frozen and thawed to damage the cell membranes, and it is then washed by water to remove components easily dissolved in water such as pigments (phycobiliprotein). Subsequently, the sample is washed using isopropanol. By stirring a large portion of sample three times, green decolorization emerges, which is gathered through filtration by applying gauze. An isopropanol-washed sample is then added into 0.1 M of NaOH solution at a temperature of 100 °C, and it is agitated at room temperature for 4 h to generate a transparent solution. This solution is then neutralized by HCl until the pH decreases to 8.0–9.0, and then it is filtered. The filtrate is concentrated using a rotating evaporator so that the solution becomes thick. To achieve deposition of the white fibrous materials, the thick solution is poured slowly into isopropanol. Afterward, to remove the added salt or generated salt from the extraction process, the fibers are re-dissolved in hot water and the solution is dialyzed against pure water for a month by changing the external water solution using regenerated cellulose membrane every day (MWCO: 14,000). The internal solution with sacran is re-thickened. The isopropanol fibrous deposit is gathered and dried in a vacuum oven (Figure 2). The sacran water solution produced does not show a specific absorption in the 220–600 nm wavelength range by using ultraviolet spectroscopy (UV-Vis). This indicates that the solution is not contaminated by proteins, nucleic acids, chromophores, and/or other chemicals that have UV-Vis absorption. The yield of sacran extraction is very high (i.e., 70% of dry *A. sacrum*) [40,41,42].

### 3.5. Characteristics of Sacran

**Figure 3:**
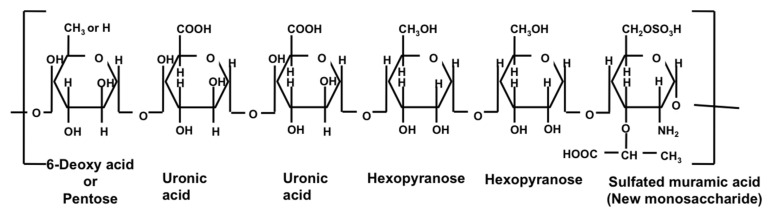
Structure of sacran [46].

Sacran is a heteropolysaccharide composed of sugar residue (galactose, glucose, mannose, xylose, rhamnose, fucose, and galacturonic acid, and glucuronic acid). It also contains traces of alanine, galactosamine, uronic acid, and muramic acid; 11% of the monosaccharide contains sulfate groups, while 22% of the monosaccharide contains carboxyl groups (Figure 3) [43]. The extraction of sacran from *A. sacrum* collected from numerous locations in Japan has generated a product that is physically similar to high-purity cotton [40,43].

Sacran characterization has been conducted on solutions containing sacran. The results of GC-MS confirmed that there are Glc, Gal, Man, Xyl, Rha, Fuc, GalA, and GlcA with composition ratios of 25.9, 11.0, 10.0, 16.2, 10.2, 6.9, 4.0, and 4.2, respectively. The T-ICR-MS of sacran with methanolysis confirmed that there is a sulfated muramic acid, a unique sugar that has only be found in sacran so far [44]. The results of FTIR confirmed that several groups consist of R-COOH, R-SO^4−^, and R-OH [45]. A GC-MS study showed that there are uronic acid–Glc or Gal (*m*/*z* 355.0869), uronic acid–uronic acid (*m*/*z* 369.0667), uronic acid–uronic acid–Gal/Glc (*m*/*z* 545.162), uronic acid–Gal–Glc–hexose (*m*/*z* 693.2089), sulfated dimethyl uronic acid (*m*/*z* 301.0239), sulfated dimethyl muramic acid (*m*/*z* 358.081), dimethylated (uronic acid–uronic acid) (*m*/*z* 435.1127), sulfated dimethylated (uronic acid–Gal–Glc) (*m*/*z* 463.0773), and Glc–Gal–N-acetyl muramic acid (*m*/*z* 630.21). FT-ICR-MS also revealed the sequence of muramic acid connectors for hexose, Glc, and Gal. These results confirmed that muramic acid is not the constituent of the cell wall but of the capsule polysaccharide of *A. sacrum* [44].

According to the analysis of Budpud et al. [47], when 0.5 wt.% of fluorescein isothiocyanate-stained sacran is observed under a super-resolution confocal microscope, it clearly shows that sacran exists in solution with a microfiber of 0.5–1 μm in diameter. Besides, this microfiber could break into particles at a submicrometer scale in NaCl solution. As Na^+^ can replace the proton of carboxylate groups, it is added to the sacran solution.

To evaluate the ability of metal ions to absorb sacran, Okajima et al. [48] conducted a test by pouring 0.5% of sacran into a metal ion solution. The result showed that sacran is able to absorb metal ions such as magnesium, calcium, manganese, iron, zinc, nickel, copper, strontium, and barium. Next, the photoreaction result of anionic gel connected with the metal cation was visualized. As a result, the gel of sacran with trivalent metal ions gradually contracts depending on the photoirradiation energy, while alginate gel as a comparison degrades [49].

A hydrogel film that has been generated by sacran at a 0.5% *w*/*v* concentration produced a film with a thickness of 0.05 mm and a 20 Wt/Wo swollen ratio, which is considered a large value [23]. Yusof et al. [28] in their research also showed that the observed flow properties of sacran solution had a low rate of shear in which its shear viscosity increased with time [50], and it was constant in an increased viscosity of 6 Pa.s. for 900 s. The increased viscosity improved with the decrease in the rate of shear. Research has found that the shear viscosity does depend on a rate of shear period of 0.8/s. Meanwhile, the shear viscosity vastly decreased in the first 10 s at a higher rate of shear of more than 1.0 s^−1^, also known as thixotropy (positive). The sacran chain was aligned with the direction of flow at low and high rates of shear. Therefore, the viscosity change completely depended on the binding time constant of inter-chain sacran. In other words, the viscosity will only increase if the rate of shear of the binding time constant is short. However, the viscosity of sacran solution has been shown to not completely depend on concentration. A sacran concentration above 0.20 wt.% was considered a neutral electric chain in a solution. Afterward, when the sacran concentration was 0.20 wt.% with the addition of NaCl (100 mL), its viscosity did not change. When the concentration increased above 0.20 wt.% with NaCl addition, the sacran chain was clearly shown to behave as a noncharged chain in a solution [41]. It has been empirically confirmed that the degree of hydrogel swelling significantly increases with the degree of dissociation. A significant increase in molar conductivity is caused by the increase in the dissociation degree of a carboxyl group. The results confirm that the free counterion of sacran contributed to the upcoming electrical conductivity upon sacran chains in a particular concentration. Moreover, sacran chains can presumably change conformation into a more compact structure by breaking down the electric charge in the sacran chain [41]. The result of XPS (X-ray photoelectron spectroscopy) at this charge suggested that with the increase in the sulfate group charge in sacran, sacran will form aggregates around the negative bias electrode [51].

## 4. Application of Sacran as Traditional Food

Sacran is a natural mega-polysaccharide obtained from cyanobacteria that has long been used by Japanese people as a food ingredient. *A. sacrum* has been proven safe for consumption, and it is also safe for use as a biomaterial for agriculture, pharmacy, and medicine [52]. It has been reported that *A. sacrum* has been consumed by the public for 300 years as a traditional food. *A. sacrum* has been cultivated since 1763 using cold cultivation methods at 20 °C with water flowing from rivers or from underground water in a traditional way [9].

This cyanobacterium is a rare cyanobacter, and it is protected in its natural habitat in Lake Ezu, Kumamoto prefecture, Japan. This alga has a very high water content compared to hyaluronic acid and xanthan gum. To eat this alga, it must first be dried and then soaked in water. It is used as a nutritious dietary supplement. Dried *A. sacrum* has a high content of vitamin B12 and other minerals such as Cu, Fe, Ca, and Mn [53].

Sacran contains several sugar residues such as galactose, glucose, mannose, and galacturonic acid. In addition, a high content of alanine, galactosamine, and muramic acid is also found in sacran [28,33]. Sugars are very important ingredients for the human body, because sugar is one of the important components that regulate the body’s physiological functions. Apart from sugar, alanine also plays an important role in boosting the body’s immune system. There are a few studies on the bioactivity of sacran in food application that can be seen in Table 1.

Japanese people have long practiced the consumption of sacran, because it is well known to treat allergies and stomach ulcers. The effect of gastric ulcers was studied by Arima et al. [55]. Sacran with a concentration of 100 mg/kg BW in mice was found to significantly reduce pain and the area of ulcers in the stomach of the tested animals. In addition, sacran was also able to reduce the weight of the tested animal. One of the possible mechanisms for this reduction is due to the high viscosity of sacran, which protects the mucous membrane of the gastrointestinal tract, thereby causing inhibition of lipid absorption by blood in the intestine. The results of this study showed a decrease in the absorption of triglycerides by rats by giving sacran at a dose of 80 mg/kg BW, where triglycerides are a factor causing obesity. Moreover, consuming sacran can also reduce oxidative stress by a mechanism that can cause a significant decrease in the levels of some pro-oxidants, such as lipids in the digestive tract, thereby reducing the further development of oxidative stress in the systemic circulation [54].

## 5. Application of Sacran in Biomedical Fields

Sacran has long been consumed by the Japanese, not only for food, but also in biomedical fields. The application of sacran in biomedical fields is described in Table 2.

### 5.1. Cancer Treatment

Cancer is a disease identified by the growth of abnormal cells. Its cause may come from both exogenous and endogenous factors that break down DNA oxidatively, a process that produces a mutation that may distract the proliferation, differentiation, and apoptosis of normal cells [73]. Cancer treatment can be conducted by using a chemotherapeutic agent. However, it must be noted that there is an efflux transporter within cells that causes resistance, which presents a challenge in formulating cancer drugs. One of the treatments is the use of selective siRNA [74,75,76].

Sacran has also been reported to be formulated for selective tumor delivery with siRNA [56,57]. Sacran has been formulated with dendrimers to target cancer. Dendrimers are spherical and porous structures that are commonly used in drug delivery for controlled delivery. Drugs are trapped via noncovalent interaction in dendrimers, or they are joined by a covalent bond in a nano-construction approach [77,78]. In previous research, a tumor-selective siRNA carrier was developed by providing a polyamidoamine dendrimer conjugated with alpha-cyclodextrin and folate-polyethylene glycol with low-molecular-weight sacran 100 (44,889 Da), 1000 (943,692 Da), and 10,000 (1,288,281 Da), such that siRNA activity transfer would be more effective. It has been tested in vitro in KB cells with a sacran concentration of 40 μg/mL but with their different molecular weight.

This confirmed that a formulation using sacran (100) increased the cellular absorption of the ternary complex, particularly on the HepG2 cell, and it was higher than that of the ternary complex with hyaluronic acid. This is due to the higher ability of sacran swelling compared to hyaluronic acid. From sacran (100), it can be inferred that cellular absorption depends on the molecular weight of sacran. Complexes formed in the presence of sacran polymer were able to escape from the efficient endosome, causing siRNA to then go to its target location. Thus, the results of the in vivo test against mouse-animal testing and the cell viability test with WST-1 have shown a significantly higher result compared to other controls [56,57].

Compared to other polymers such as alginates, sacran shows hydrophobization and the phenomenon of insolubilization in response to ultraviolet light irradiation. This photoresponsive mechanism was investigated in the corresponding gels formed by interaction with metal ions. Sacran gels contracted and alginate gels degraded. The photo-shrinkage of the sacran gels may be attributed to the hydrophobization of uronic acid based on photo-decarboxylation. This will increase the effectiveness of drug release for cancer delivery if radioactive ions are used [24,79]. Sacran in a liquid crystalline hydrogel form is a biocompatible material with L929 cells of mouse fibroblast. As a result, there is no effect of toxicity prevention, growth increases, and cell proliferation [72].

### 5.2. Wound Dressing

A wound is defined as a skin layer (mucosa) disorder due to physical or thermal damage. It consists of 4 (four) phases, including coagulation, hemostasis, inflammation, proliferation, and remodeling [80,81]. The application of a wound dress plays a crucial role in wound healing and infection prevention [82,83]. Therefore, many materials have been developed as a better wound dressing. Hydrogel film is one that drew people’s attention as a wound dressing. Hydrogel contains both synthetic and natural polymers. One of the natural polymers that has been developed as a wound dress is sacran [61].

Sacran has been developed as a wound dressing material as its activity in absorbing exudate and its characteristics are better for human skin in the wound healing process. This is due to its ability to expand and its high porosity [63]. The swelling degree of crosslinked sacran gel in pure water increases with pH. In the salt solution, the swelling degree does not change significantly. Moreover, in the acidic condition, with a combination of chitosan and benzaldehyde-terminated Pluronic^®^F127 hydrogel, intramolecular electrostatic repulsion occurs and increases the swelling degree by improving the hydrophobicity of this hydrogel [83].

The maximum swelling degree of sacran gel in pure water is 6100, which is equivalent to water absorption of a spontaneously bonded sacran, measured with the tea bag method. This swelling ratio might be obtained from a large mesh of long sacran chain tissues [84,85]. A film of sacran hydrogel could be swollen to 70 times, which is potentially thicker than its width. If the molecular weight of sacran is decreased, it will cause a slight anisotropy on swelling [86].

Sacran for a wound dressing has been formulated in a hydrogel. The methods used in obtaining this sacran hydrogel have been developed by applying the freeze-drying technique [86], solvent-casting technique [23], and casting technique [61]. The sacran formulation in hydrogel presents anisotropic properties. To produce sacran hydrogel with superanisotropy, it can be applied by using a crosslinking agent in divinyl sulfonate with a two-step crosslinking method [30]. Another crosslinking agent that has been applied is l-lysin, which can increase the absorption of metal ions compared to sacran hydrogel without crosslinking. As crosslinking agents can break the sacran structure, the metal ions will gradually diffuse to the inner structure and the hydroxyl groups of sacran will be available to effectively chelate the absorbed metal ions [87]. According to Zhang et al. [88], a low density of crosslinker will allow water molecules to enter the hydrogel network; therefore, it can break the hydrogel structure and cause diffusion.

By conducting a sacran hydrogel test upon wound dressing and relying on its good improvement with a 0.5% (*w*/*v*) sacran hydrogel concentration, sacran hydrogel in an in-vivo test could dress the wound on the fifteenth day with more than 80% of wound closure [60]. Other research by Wathoni et al. and Motoyama et al. found that the wound was 100% closed on the twelfth day, meaning that it worked much better than the film of sodium alginate hydrogel. It was caused by the ability of sacran to absorb the wound exudate so that it could prevent some enzymes that slow down the wound healing [23,59]. The mechanism of sacran in absorbing the exudate is needed in the wound area to improve the wound healing process [89].

Sacran has long been formulated in hydrogel film by adding cyclodextrin (alpha and gamma) that may increase the film’s ability to expand [58]. In research by Wathoni et al. [90], the sacran hydrogel was formulated with epidermal growth factor (EGF) for wound healing. It indicated that EGF addition causes a lower swelling ratio than that without EGF. However, its tensile strength was larger than that of sacran with EGF. By observing cell migration using NH3T3 cells, it has been identified that sacran with EGF changed significantly in 18 h. Thus, the existence of EGF in sacran hydrogel has been identified to accelerate wound healing as EGF may bond with heparin in hyaluronic hydrogel or collagen. Another study of sacran activity as a wound healing agent was carried out by Goto et al. [62]. The sacran formulation added to the tetrahydrocurcumin-HP-β-CD complex showed that this complex improved wound healing faster than the controls on days 7, 10, and 14. This is due to the addition of active ingredient tetrahydrocurcumin, which can improve wound healing.

The main mechanism of sacran in wound healing is the absorption of wound exudate caused by its good porosity, thus preventing enzymes that can slow wound closure. In addition, sacran can also maintain skin moisture, thereby increasing the rate of wound healing. The presence of a sulfonated group that is also an anticoagulant agent in sacran does not affect its activity; this has been proven by the results of previous studies. The research using polymers containing sulfur also showed good wound healing results. The hemostatic mechanism is due to the stable structure of the hydrogel shaft, thus supporting platelet aggregation and erythrocyte capture for blood clotting and for generating a stable wound to achieve quick hemostasis [91]. This is the same characteristic from sacran, which has good porosity [63].

### 5.3. Anti-Allergy

An allergy is known as a hypersensitivity reaction that occurs when the immune system overreacts to an antigen [92]. Antiallergy refers to the ability to prevent allergy reactions in particular stages and to control allergic inflammation symptoms [93]. Sacran as a compound generated from river algae, namely *A. sacrum*, is one of the polysaccharides from cyanobacteria that is preferable in preventing dry skin of humans by reducing transepidermal water loss [65]. Sacran has also been reported to have anti-allergic activity to atopic dermatitis. Atopic dermatitis is a skin disease identified with heterogeneous pathogenesis, including skin barrier disfunction, allergy, and pruritus. This disease is triggered by the immune system to antigens and irritants, and is frequently linked to allergy type 1, allergic rhinitis, or asthma [94,95].

β-Hexosaminidase that is released from activated mast cells is responsible for the allergic inflammation response that is related to the atopic dermatitis condition. Therefore, in investigating the activities of sacran antiallergic effects, the evaluation of sacran inhibition effects on β-hexosaminidase release in RBL-2H3 cells was carried out. It is currently known as a mouse mucosal mast cell analog. Sacran significantly reduces the β-hexosaminidase release to 70%, in comparison with its control. This result has confirmed that sacran can inhibit the allergic inflammation response [67].

Motoyama et al. [63] tested sacran against an atopic dermatitis mouse model with itchiness as a symptom [64]. After inducing mice with a hapten, including dinitrofluorobenzene (DNFB), it indicated that sacran with a 0.05% concentration can decrease the itchiness of an atopic dermatitis mouse. In another study, using sacran in different concentrations against a mouse model that has been induced by DME (similar to atopic dermatitis), it was shown that 0.2% and 1% sacran concentrations can decrease the severity of skin lesions in an allergic mouse in the first-week treatment and can inhibit the symptoms [96].

Other research also investigated the anti-allergic effect of topical sacran to several atopic dermatitis patients. The results confirmed an improvement of skin symptoms in the fourth week of treatment. The effects of sacran on patients were obtained from the results of a questionnaire with 13 questions. On average, a score uplift less than 2 showed an exacerbation of atopic dermatitis, while a score more than 2 showed the skin progress of patients. Besides, it could be identified with their sleeping difficulty status and feeling itchy as a symptom. These two results were eventually shown to decrease in the fourth week after using sacran topically. Thus, it can be inferred that sacran can enhance patient condition [63].

### 5.4. Anti-Inflammation

Inflammation or inflection is a biological response of the immune system that can be triggered by some factors, including pathogens, damaged cells, and toxic compounds [97]. Many reports have revealed that sacran can reduce the inflammation response on atopic dermatitis. Atopic dermatitis is one of the skin diseases identified with inflection, pruritus, and chronic eczema lesion [67]. Anti-inflammatory therapy for atopic dermatitis is targeting cytokine receptors such as interleukin [98,99,100].

It has been reported that sacran has anti-inflammatory and antiallergic activity on atopic dermatitis. Some research conducted an anti-inflammatory test against a mouse model induced by DNFB (2.4-dinitrofluorobenzen). It has been found that sacran with 0.01% (*w*/*v*) and 0.05% (*w*/*v*) concentrations were able to reduce mRNA levels of inflammatory cytokine and chemokines, such as MCP-1, TNF-α, IL-1β, and dan IL-6 [67,68]. Sacran can also reduce foot swelling and neutrophil infiltration in mouse ear tissues. Moreover, sacran can inhibit edema of a mouse’s ear induced with TPA (12-O-tet-radecanoylphorbol-13-acetate) [62]. Sacran can also inhibit foot swelling and neutrophil infiltration in edema of a mouse’s foot induced with carrageenan. In addition, sacran can significantly inhibit edema of a mouse’s ear induced with 12-O-tetradecanoylphorbol-13-acetat (TPA) or carrageenan and reduce mRNA expression levels of COX-2 and pro-inflammatory cytokines such as TNF-α, IL-1β, and IL-6 [33].

### 5.5. Other Application

Sacran can be formulated as cosmetics as its larger particles have made it difficult to penetrate the skin, so it can remain on the surface of the skin [70]. Human skin can heal itself and may experience the external violent ambiance or stress changes [101]. One of the skin problems that many people experience is dry skin. Skin dryness can be identified with low hydration or when the trans-epidermal water loss level is high. Sacran can inhibit the excessive evaporation of water from the skin. Sacran can also play an important role in managing optimal skin conditions so that keratinocyte may develop through its differentiation [71]. Furthermore, sacran can weaken some effects of external stimuli that damage the skin by applying a wrapping stimuli system within its matrix [70], due to the existence of polyol groups in sacran [32]. Sacran can also retain liquid so that it may prolong drug release effects. Thus, sacran can be formulated with various drugs to prolong its release [62,102].

Sacran that has been formulated topically is retained in the skin surface or stratum corneum. This polysaccharide can repair oxidative stress caused by tobacco smoke through protecting the skin surface. This protection emerges throughout the shielding effects of benzopyrene compound (BaP) and aldehyde contained in tobacco. Sacran can inhibit the increase in P4501A1 mRNA cytochrome regulation that is a xenobiotic metabolizers enzyme induced by BaP and other responses to tobacco smoke in HaCaT keratinocytes [103].

Sacran as the extraction result of *A. sacrum* can reduce irritations in skin damage or suppress gastric ulcers pain induced by NSAID and HCl/EtOH. Sacran can be used orally or topically to heal skin irritation and inhibit gastrointestinal ulcers [55]. Some reports reveal that sacran consumption can reduce prooxidant levels significantly, such as the uremic toxin in the digestive tract. Thus, it can inhibit the further progress of oxidative stress in systemic circulation and can decrease kidney damage effectively as well [104]. Sacran has been clinically tested topically in improving skin problems caused by an impaired epidermal barrier. This test was carried out by giving serum formulated with sacran to volunteers. The result showed that sacran can inhibit the excessive evaporation of water from the skin; sacran could also play an important role in providing optimal skin conditions for keratinocytes, and it can improve the maturation of corneocytes [27,71].

Another application of sacran is genetic engineering. By observing sacran’s ability in genetic engineering, it has been shown that sacran is a potential biomaterial to scaffolding tissue engineering as sacran can directly stick on the surface of animal tissue cells in an extracellular matrix [72]. Sacran is also capable of controlling mechanical properties, and cell adhesion leads to applications for genetic engineering [37].

## 6. Conclusions and Outlook

Sacran is a natural polysaccharide derived from the algae called *Aphanothece sacrum* (*A. sacrum*), which is known as a good material to be applied in biomedical fields. Several researchers have reported the extraction and isolation process of *A. sacrum* using base and acid to remove minerals contained therein. Sacran has a negative charge in water, and due to its high viscoelasticity and water retention, it can protect the skin surface. Thus, it has been applied in many cosmetics and medical products. Sacran has been formulated in several pharmacological activities, such as wound dressings, anti-inflammatory agents, antiallergic agents, anti-cancer polymers, an additive in cosmetics, and inhibitor agents for gastric and intestinal ulcers.

Sacran can be used as a beneficial material either for medicine or an additive in some formulations. The biomedical fields still lack the research that discusses the development of sacran as sacran is a potential material for medicine and a good additive that can be developed.

## Figures and Tables

**Table 1:** The bioactivity studies of sacran in food application.

Bioactivity	In Vivo Test	Ref.
Reduce body weight	Tested on male mice (160–180 g) for 8 weeks	[54]
Reduce oxidative stress	Tested on male mice (160–180 g) for 8 weeks	[54]
Painkiller and reduce gastric ulcers	Tested on mice induced with HCl/EtOH for gastric ulcer	[55]

**Table 2:** Sacran activity testing in biomedical fields.

Application in Biomedical Fields	Testing Object	Test Type	Ref.
Cancer Delivery	Polyamidoamine conjugates alpha-cyclodextrin and phosphate-polyethylene glycol with low-molecular-weight sacran for selective siRNA delivery	In Vitro and In Vivo	[56,57]
Wound Dress	Sacran hydrogel	In Vitro and In Vivo	[23,58,59]
	γ-Cyclodextrin addition to sacran hydrogel film	In Vitro and In Vivo	[58]
	Curcumin addition to 2-hydroxypropil-γ-cyclodextrin on hydrogel film	In Vitro and In Vivo	[60]
	Sacran hydrogel film with keratinocyte growth factor	In Vitro	[39]
	Epidermal growth factor (EGF) in sacran hydrogel film as the increased fibroblast migration	In Vitro	[61]
	HP-βCD complex in freeze dried Sac/SDACNF	In Vivo	[62]
Anti-allergy	Topical sacran in mouse model induced by 2,4-dinitro-1-fluorobenzene	In Vivo	[63]
	Topical sacran in mouse model induced by DME-	In Vivo	[64,65]
	Topical sacran for atopic dermatitis	In Vivo	[66]
Anti-inflammation	Sacran for atopic dermatitis in mouse induced by 2,4-dinitro-1-fluorobenzene	In Vivo	[67,68]
	Sacran for atopic dermatitis in mouse induced by 2,4-dinitro-6-fluorobenzene	In Vivo	[44]
	Sacran and carrageenan induced in TPA-induced mouse ear	In Vivo	[33]
Others	Effectiveness of sacran on air pollution	In Vitro	[69]
	Effectiveness of sacran to prevent skin evaporation	In Vitro	[70]
	Improve the maturation of corneocytes	Clinical study	[27,71]
	Genetic engineering	In Vitro	[37,72]
